# Molecular Insights Into Lysyl Oxidases in Cartilage Regeneration and Rejuvenation

**DOI:** 10.3389/fbioe.2020.00359

**Published:** 2020-04-30

**Authors:** Weiping Lin, Liangliang Xu, Gang Li

**Affiliations:** ^1^Department of Orthopaedics and Traumatology, Stem Cells and Regenerative Medicine Laboratory, Li Ka Shing Institute of Health Sciences, The Chinese University of Hong Kong, Prince of Wales Hospital, Hong Kong, China; ^2^The First Affiliated Hospital of Guangzhou University of Chinese Medicine, Lingnan Medical Research Center, Guangzhou University of Chinese Medicine, Guangzhou, China; ^3^MOE Key Laboratory for Regenerative Medicine, School of Biomedical Sciences, The Chinese University of Hong Kong, Hong Kong, China

**Keywords:** lysyl oxidase, cartilage, hypoxia-inducible factor, copper, transcription activity, regeneration, rejuvenation

## Abstract

Articular cartilage remains among the most difficult tissues to regenerate due to its poor self-repair capacity. The lysyl oxidase family (LOX; also termed as protein-lysine 6-oxidase), mainly consists of lysyl oxidase (LO) and lysyl oxidase-like 1-4 (LOXL1-LOXL4), has been traditionally defined as cuproenzymes that are essential for stabilization of extracellular matrix, particularly cross-linking of collagen and elastin. LOX is essential in the musculoskeletal system, particularly cartilage. LOXs-mediated collagen cross-links are essential for the functional integrity of articular cartilage. Appropriate modulation of the expression or activity of certain LOX members selectively may become potential promising strategy for cartilage repair. In the current review, we summarized the advances of LOX in cartilage homeostasis and functioning, as well as copper-mediated activation of LOX through hypoxia-responsive signaling axis during recent decades. Also, the molecular signaling network governing LOX expression has been summarized, indicating that appropriate modulation of hypoxia-responsive-signaling-directed LOX expression through manipulation of bioavailability of copper and oxygen is promising for further clinical implications of cartilage regeneration, which has emerged as a potential therapeutic approach for cartilage rejuvenation in tissue engineering and regenerative medicine. Therefore, targeted regulation of copper-mediated hypoxia-responsive signalling axis for selective modulation of LOX expression may become potential effective therapeutics for enhanced cartilage regeneration and rejuvenation in future clinical implications.

## Introduction

The lysyl oxidase family (LOX; also termed as protein-lysine 6-oxidase) has been traditionally defined as cuproenzymes that are essential for stabilization of extracellular matrix (ECM), particularly cross-linking of collagen and elastin (Rucker et al., 1998; Kagan and Li, 2003). LOX mainly comprises of five members that were originally considered copper-dependent amine oxidases, including lysyl oxidase and lysyl oxidase-like 1-4 (LOXL1-LOXL4), is a copper-containing amine oxidase belonging to a heterogeneous family of enzymes, which catalyzes oxidative deamination of the amino group in certain lysine and hydroxylysine residues of collagen molecules for stabilization of collagen fibrils (Kagan and Li, 2003). To date, LOX has been demonstrated to regulate a diverse range of cellular processes and biological functions (Smith-Mungo and Kagan, 1998), as well as certain pathogenesis of various diseases, particularly fibrotic diseases, ischemic cardiovascular diseases, and cancer progression, which is mainly mediated by ECM remodeling and elastogenesis (Schmelzer et al., 2019), epithelial-mesenchymal transition and intracellular signaling (Lopez et al., 2010; Busnadiego et al., 2013; Cox et al., 2013, 2015; Klingberg et al., 2013; Rimar et al., 2014; Schmelzer et al., 2019). The LOX has been demonstrated as crucial contributors for normal embryonic development of various tissue and organ systems, including cardiovascular (Martinez-Gonzalez et al., 2019), and respiratory systems (Maki et al., 2002; Maki, 2009; Maki et al., 2005), as well as essential for normal physiological and cellular properties, such as sprouting angiogenesis of endothelial cells (Lucero and Kagan, 2006; Bignon et al., 2011).

Cartilage is an avascular collagen-abundant tissue. Unlike bone, cartilage seems to lack efficient self-reparative/regenerative capacity, making arthritis common and costly, affecting the well-being and quality of life of millions of people worldwide (Huey et al., 2012; Mobasheri and Batt, 2016; Deng et al., 2019). Inflammatory arthritides, such as rheumatoid arthritis, and psoriatic arthritis, are among the most challenging auto-immune diseases and health problems worldwide (Mobasheri and Batt, 2016; Flores et al., 2019; Zhou B. et al., 2019). Of note, osteoarthritis (OA) is the most common form of arthritis, which is one of the most prevalent chronic immune diseases. OA is characterized by articular cartilage degeneration, subchondral bone remodeling, osteophyte formation and synovial changes (Yuan et al., 2014). OA is a multifactorial disease and various risk factors of OA have been reported, such as obesity (Aspden, 2011), body mass (Messier et al., 2005), and aging (Lotz and Loeser, 2012). Until now, the etiology and pathophysiology of OA have not been well documented. Various treatments, such as cellular therapies (Fu et al., 2014; Lee and Wang, 2017; Lin et al., 2017; Teo et al., 2019; Xu et al., 2019), administration of certain drugs or chemicals (Zhang et al., 2016, 2019; Yao et al., 2019), therapeutic surgeries (Chen Y. et al., 2015), and bio-fabrication approach (Tatman et al., 2015; Onofrillo et al., 2018; Lee et al., 2019), have been intensely studied and tested in various preclinical studies and clinical trials during recent decades (Lee and Wang, 2017; Zhang et al., 2019). However, several hurdles are required to be addressed for therapeutic optimization before clinical translation. And currently there are no effective clinical options treating OA (Mobasheri and Batt, 2016; Chen et al., 2018; Griffin and Scanzello, 2019).

Cross-linking is essential for the stabilization and mechanical support of collagen networks within native cartilage. Of note, the formation of lysine-derived, covalent pyridinoline (PYR) cross-links relies on the enzyme LOX, which is hypoxia-response element-directed upregulation during HIF1-transcriptional activation (van Vlimmeren et al., 2010; Gao et al., 2013). The activity of LOX is regulated by proteolytic cleavage of the LOX pro-peptides. Importantly, the activity of LOX is also mainly dependent on the presence of copper (Wang et al., 1996). The LOX family plays central roles in the musculoskeletal system, such as tendon (Danielsen, 1982; Robinson et al., 2005; Marturano et al., 2013), ligaments (Makris et al., 2014), and cartilage (Iftikhar et al., 2011; Makris et al., 2014).

Of note, copper, an important co-factor of various chaperones and enzymes, is vital for maintenance of integrity and homeostasis of cartilage tissues. However, until now, the underlying detailed mechanisms remain elusive. In the current review, we summarized the advances of LOXs family in cartilage homeostasis and regeneration, including embryogenesis, and potential involvement during pathophysiology of arthritis, as well as copper-mediated activation of LOXs and hypoxia-responsive signaling axis during recent decades, the main molecular modulation signaling network controlling LOXs expression, which is promising for potential clinical implications of cartilage regeneration in regenerative medicine and tissue engineering. Also, we propose potential links between the LOXs family and aging-related chronic inflammation and cartilage degeneration. Modulation of certain the LOXs family members may become a promising therapeutic approach for cartilage regeneration.

## Lysyl Oxidases in Cartilage Functioning

Endochondral ossification is one of the two main forms of skeletal formation during embryogenesis. Mesenchymal chondroprogenitor cells differentiate into chondrocytes through cellular condensation processes, which are then surrounded by an abundant layer of extracellular matrix, including type II, IX, and XI collagens, which is the characteristics of cartilage (Mendler et al., 1989).

**FIGURE 1:**
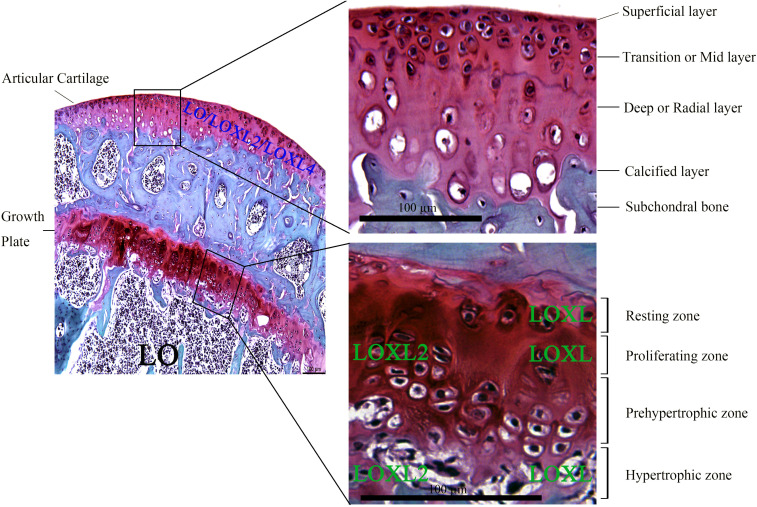
Histology of growth plate and articular cartilage through Safranin O-Fast green staining of the proximal tibia from adult C57BL/6 mice. Mature articular cartilage is mainly comprised of four zones based on histologic features: superficial layer, transition or mid (middle) layer, deep or radial layer, and calcified layer that is lined by subchondral bone. Growth plate is mainly characterized by several morphologically distinct zones, including resting zone, proliferating zone, prehypertrophic zone, and hypertrophic zone. LO is mainly expressed in marrow cavity, while LOXL2 expression is mainly localized in proliferating and hypertrophic zones of growth plate. LOXL was mainly found on chondrocytes of resting, proliferating and hypertrophic zones of growth plate. The expression of LO, LOXL2, and LOXL4 was descended from superficial to deep layers of articular cartilage. Lysyl oxidase: LO; lysyl oxidase like-2: LOXL2; lysyl oxidase like-4: LOXL4; Lysyl oxidase-like enzymes: LOXL. Scale bar: 100 μm.

Generally, aging-induced cartilage degeneration in arthritis is becoming increasingly prevalent, which is accompanied with the changes in the components of ECM of cartilage (Loeser et al., 2016; Varela-Eirin et al., 2018). The expression of LO and LOXLs has been detected in chondrocytes near the joint cavity undergoing appositional growth, as well as in the epiphyseal plate of femur undergoing endochondral ossification. Strong expression of LO was observed in marrow cavity. And the localization of LOXL was mainly detected in chondrocytes of the reserve, proliferating cartilage, and hypertrophic zones, suggesting their vital functions during cartilage embryogenesis and potential roles for the normal function of adult cartilage (Hayashi et al., 2004; Thomassin et al., 2005). Further studies have indicated the expression of LO, LOXL2, and LOXL4 on chondrocytes of articular cartilage layers (Huang et al., 2010). Also, expression of LOXL2 has been detected in proliferating and hypertrophic chondrocytes of normal growth plate *in vivo* (Iftikhar et al., 2011). The general histologic structure of joint cartilage (mainly including growth plate and articular cartilage) has been presented in Figure 1.

**FIGURE 2:**
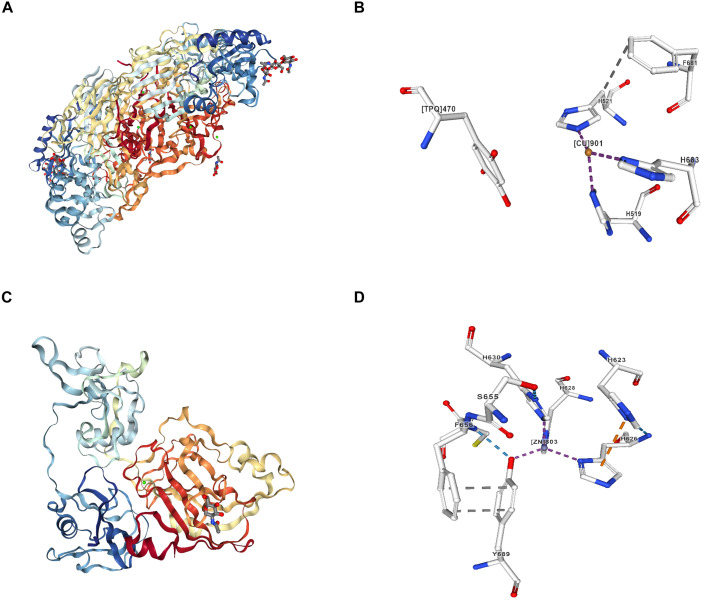
Crystal structures and ligand interactions of copper-containing amine oxidase and human lysyl oxidase-like 2 (http://www.rcsb.org/). **(A)** Copper- containing amine oxidase extracted from bovine serum (BSAO) was crystallized and its three-dimensional structure at 2.37A resolution. The biological unit of BSAO is a homodimer, formed by two monomers related to each other by a non-crystallographic 2-fold axis. Each monomer is composed of three domains. **(B)** Ligand interaction of copper iron [(CU)901] in copper-containing amine oxidase extracted from BSAO. **(C)** Crystal structure of human lysyl oxidase-like 2 (hLOXL2) at 2.4-Å resolution. **(D)** Ligand interaction of zinc iron ([ZN]803:A) in hLOXL2. The copper-binding site of hLOXL2 is occupied by zinc, which blocks lysyl tyrosylquinone (LTQ) generation and the enzymatic activity of hLOXL2. The LTQ precursor residues in the structure are distanced by 16.6 Å, corroborating the notion that the present structure may represent a precursor state and that pronounced conformational rearrangements would be required for protein activation.

Notably, the activity of LOX is of pivotal importance for maintaining the tensile and elastic features of connective tissues in the musculoskeletal (Weiner and Traub, 1992; Herchenhan et al., 2015), cardiovascular and pulmonary systems (Ohmura et al., 2012; Nave et al., 2014). In cartilage, LOX is capable of modification of amino acids lysine and hydroxylysine into covalent PYR cross-linking (i.e., heterotypic collagen II/IX/XI) (Eyre et al., 2004), in particular the most abundant type of cross-links in native articular cartilage, which is tightly correlated with the tensile properties of native articular cartilage (Williamson et al., 2003; Eyre et al., 2008). Nevertheless, inactivation of LOXs induced by copper metabolic disorder or gene mutation would lead to dysfunction of connective tissues and collagen-containing organs (Kuivaniemi et al., 1982; Maki et al., 2002). To date, the discovery of crystal structures of copper-containing amine oxidase and lysyl oxidase-like 2 has been reported in the current literature (Figure 2) (Duff et al., 2003, 2004; Lunelli et al., 2005; Zhang X. et al., 2018).

To date, the LOX family has been reported essential for cartilage maturation, chondroprotection, and homeostasis maintenance of cartilage. LO and LOXL-3b have been demonstrated crucial for cartilage maturation during zebrafish development, respectively (Reynaud et al., 2008; van Boxtel et al., 2011). Further, studies have indicated the upregulation of LOXL2 in OA cartilage in response to injury, which may be considered as a naturally protective response that promotes anabolism while inhibiting specific catabolic response during OA pathophysiology (Alshenibr et al., 2017; Bais and Goldring, 2017). Likewise, a recent study further confirmed that systemic adenovirus-delivered LOXL2 expression or LOXL2 genetic overexpression both exhibited chondroprotective effects through inhibition of catabolic factors and IL-1β-induced NF-κB signaling in mice (Tashkandi et al., 2019). Also, Matrigel constructs of human chondrocytes from the knee joint and TMJ implanted in nude mice showed enhanced anabolic responses after LOXL2 transduction, including increased expression of sex determining region Y-box containing gene 9 (SOX9), aggrecan (ACAN), and COL2A1, whilst reduced the levels of extracellular matrix (ECM)-degrading enzymes matrix metalloproteinases and inhibited chondrocyte apoptosis (Alshenibr et al., 2017). Therefore, LOX-mediated collagen cross-links are essential for the functional integrity of articular cartilage and cartilage homeostasis. Appropriate modulation of the expression or activity of certain LOXs family members selectively may become potential promising strategy for cartilage repair.

The components of extracellular matrix are vital for the maintenance of phenotype and function of chondrocytes (Shi et al., 2017; Li et al., 2018; Zhang et al., 2020). The expression of a novel LOX-related gene, named LOXC, has been detected in cartilage *in vivo*, which modulates the formation of collagenous extracellular matrix (Ito et al., 2001). A series of further studies have confirmed LOX as a key enzyme responsible for the formation of collagen cross-links. Furthermore, hypoxia-induced endogenous LOX expression has been applied in the repair of *de novo* multiple musculoskeletal tissues (i.e., cartilage, meniscus, tendons, and ligaments) as important regenerative strategies, which is mainly mediated through mechanisms of hypoxia-induced enhanced PYR crosslinking and increased tensile properties of collagen-rich tissues (Makris et al., 2014).

Simultaneously, studies have demonstrated that combined treatment of copper sulfate and hydroxylysine would additively or synergistically enhance collagen cross-linking in engineered articular cartilage, improving the tensile and biomechanical properties of the neocartilage (Makris et al., 2013). LOXL2 promotes chondrogenic differentiation through regulation of SOX9 and SNAIL (Iftikhar et al., 2011). Also, LOX activity has been reported vital for phenotypic modulation of chondrocytes (Farjanel et al., 2005). Therefore, modulation of endogenous LOX activity or expression selectively has been demonstrated effective for promotion of tensile properties and cross-linking of cartilage, as well as phenotypic control of chondrocytes, which appears as promising clinically applicable approaches of regenerative medicine and tissue engineering (Makris et al., 2014; Hadidi et al., 2017) (Table 1).

**TABLE 1:** Therapeutic approaches for enhanced cartilage regeneration through LOXs modulation.

**Authors**	**Source/Species**	**Detailed Treatments**	**Experimental Model**	**Therapeutic Outcomes**	**Mechanisms**
Makris et al., 2014	Calves	Continuous hypoxia conditioning or exogenous LOXL2 administration	Trochlea groove cartilage and knee meniscus explants	Enhanced neocartilage formation and functional properties	Increased collagen cross-linking
Tashkandi et al., 2019	Mice	Intra-articular injection with MIA (monosodium iodoacetate) or intraperitoneal injection of adenovirus vector (Adv)-RFP-LOXL2	MIA-induced OA in LOXL2-overexpressing transgenic mice or Cho/+ mice injected with Adv-RFP-LOXL2	Systemic LOXL2 adenovirus or LOXL2 genetic overexpression in mice protected against OA	Inhibition of IL-1β-induced phospho-NF-κB/p65 and MMP13 expression; upregulation of anabolic genes
Makris et al., 2013	Juvenile bovine knee joints	0.0016 mg/ml copper sulfate and 0.146% mg/ml hydroxylysine either or in combination	Chondrocytes-self-assembly-tissue culture constructs	Synergistic tensile properties in combination of copper sulfate and hydroxylysine-treated group	Enhanced PYR cross-links
Hadidi et al., 2017	Bovine hind limbs taken from skeletally immature calves	Exogenous LOXL2 administration along with copper and free hydroxylysine	Culture constructs: primary articular chondrocytes and meniscus cells seeded in non-adherent agarose wells	Enhanced tensile properties of and near-native tissue values in terms of glycosaminoglycan content in LOXL2-treated constructs	Increased collagen and PYR cross-links
Lin R. et al., 2019	New Zealand white rabbits	Copper-incorporated bioactive glass-ceramics (Cu-BGC)	Model of osteochondral defects with a diameter of 5 mm	Facilitated the regeneration of cartilage and osteochondral interface significantly by Cu-BGC treatment	Activation of HIF-1 signaling and inhibition of inflammatory response via inducing an anti-inflammatory M2 phenotype in macrophage
Alshenibr et al., 2017	Human	*In vivo* implantation of human articular and temporomandibular joints (TMJ) chondrocytes in nude mice; expression detection in human tissue sections	Human knee and hip joints and TMJ	Upregulated expression of LOXL2 in OA cartilage	A protective response that promotes anabolism while inhibition of specific catabolic responses (promoted specific chondrogenesis in implants lacked fibrosis and mineralization)
Iftikhar et al., 2011	Not available	Induction of chondrogenic differentiation	ATDC5 cell line	Expression of LOXL2 in ATDC5 chondrogenic cells and LOXL2 promoted ATDC5 chondrogenic differentiation	Through regulation of SOX9 and SNAIL

### Regulation of Hypoxia-Inducible Factor-1 in Chondrogenesis and Cartilage Homeostasis

The physiological oxygen tension between 2%∼9% in healthy individuals, which is termed as ‘physiologic normoxia’ (Simon and Keith, 2008). Generally, hypoxia-inducible factor 1 (HIF-1) is expressed in a variety of organs and tissues in healthy mammalians under physiologic normoxic conditions, including brain, kidney, liver, heart, and cartilage (Stroka et al., 2001; Coimbra et al., 2004). Articular cartilage is residing in a hypoxic microenvironment of avascular hypoxic zone *in vivo* under normal physiological conditions, ranging from 7∼10% oxygen tensions in the superficial zone, and 1% oxygen in the deep zones (Ferrell and Najafipour, 1992). Hypoxia-inducible factor 1α (HIF-1α) have been demonstrated essential for cartilage maturation (Duval et al., 2009; Stegen et al., 2019). Also, local activation of HIF-1α is necessary for survival and homeostatic function of chondrocytes, as well as normal joint development (Aro et al., 2012; Long, 2019). Thus, hypoxia and HIF-1 have been exploited to modulate chondrocyte phenotype and represent an efficient approach to improve cell properties before implantation for cartilage repair.

Notably, HIF-1 is of pivotal significance for survival and growth arrest of chondrocytes during cartilage development, as well as cartilage homeostasis of osteoarthritic cartilage (Pfander et al., 2006; Gelse et al., 2008). HIF-1 is conducive to the maintenance of chondrogenic specific markers (SOX9, type II collagen, and aggrecan) and inhibition of cartilage hypertrophy (Duval et al., 2012). HIF-1 has been demonstrated as a positive regulator of SOX9 activity, which is required for chondrogenesis and synthesis of cartilage ECM (Robins et al., 2005; Zhang et al., 2011). Nevertheless, dysregulation of HIF-1α signaling axis would lead to skeletal dysplasia by interfering with cellular bioenergetics and biosynthesis (Stegen et al., 2019). A recent study has indicated the increased expression of genes involved in matrix degradation, hypoxia-responsive, and inflammatory signaling in damaged cartilages comparing with healthy counterparts through whole genome microarray analysis, suggesting potential involvement of HIF-1α during the progression of OA pathophysiology (Yudoh et al., 2005; Aşık et al., 2019), which may be a natural protection response of the body since previous studies have demonstrated chondroprotection efficacy through activation of HIF-1 signaling axis (Gelse et al., 2008; Maes et al., 2012; Lin R. et al., 2019). Further comprehensive studies elucidating detailed roles of HIF-1α in certain stages of OA pathogenic progression, as well as detailed mechanisms on hypoxia-induced LOXs expression and activity, require further elucidation. Therefore, appropriate modulation of transcriptional activity of HIF-1α may become a potential feasible therapeutic approach for cartilage regeneration.

### Chondroprotective Effects of Copper in Cartilage

Copper, an essential redox-active trace element, which is essential for most aerobic organisms (Tapiero et al., 2003; Solomon et al., 2014). Simultaneously, copper functions as a co-factor of various proteins and enzymes, including cytochrome C, superoxide dismutase, tyrosinase, ascorbate oxidase, lysyl oxidase, and amine oxidase, exhibiting diverse fundamental cellular functions in normal physiology, including energy generation, iron acquisition, oxygen transportation, cellular metabolism, peptide hormone maturation, blood clotting, neurotransmitter biosynthesis, and intracellular signal transduction (Huffman and O’Halloran, 2001; Hamza and Gitlin, 2002; Kim et al., 2008; Turski et al., 2012; Grubman and White, 2014; Wang et al., 2018; Miller et al., 2019). Generally, copper is able to exist in two oxidation states in the body of mammalians: Cu^+^ and Cu^2+^ (Lin and Kosman, 1990; Pushie et al., 2007; Solomon et al., 2014). The trafficking of copper into specific intracellular targets is delivered by metallochaperones (Hamza et al., 2001; Puig and Thiele, 2002; Chen G. F. et al., 2015). And there is no free copper in the cytoplasm under normal physiological conditions (Rae et al., 1999).

In general, moderate copper levels are essential for normal growth, development, health, such as the normal functioning of innate immune system (Djoko et al., 2015; Bost et al., 2016), and bone health (Eaton-Evans et al., 1996; Qu et al., 2018). Of note, copper is also vital for maintenance of integrity and homeostasis of cartilage tissues. Copper metabolic disorder correlates closely with ischemic cardiovascular diseases (Jiang et al., 2007; Kim et al., 2010), embryonic and neonatal abnormalities, and anemia (Cartwright and Wintrobe, 1964; Jensen et al., 2019), as well as the onset of osteoarthritis (Scudder et al., 1978; Yazar et al., 2005).

**FIGURE 3:**
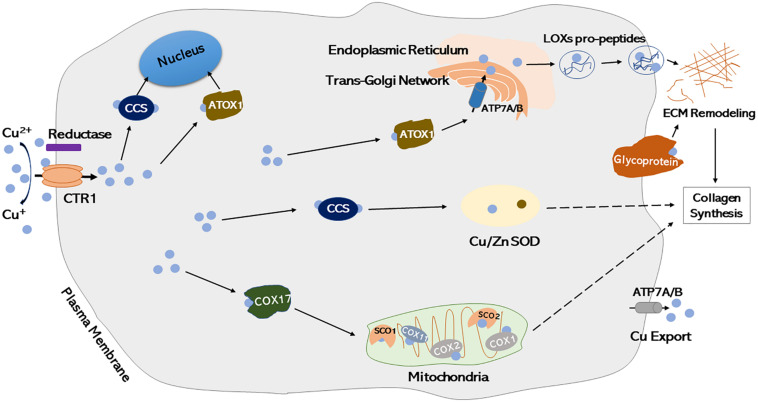
Proposed cellular models of intracellular copper trafficking in chondrocytes. Cu^2+^ is transformed to Cu^+^ by a putative metalloreductase, which then enters the cell through plasma membrane importer CTR1. Once inside the cell, copper is handed off to copper chaperones. CCS incorporated copper into the cytosolic protein Cu/Zn-SOD. ATOX1 delivers copper into secretory compartments of Golgi and Endoplasmic Reticulum through ATP7A/B. Copper can be incorporated into copper-dependent proteins, such as LOXs pro-peptides (secreted forms of LOXs), which is involved in ECM remodeling. Once inside the intermembrane space of mitochondria, copper is handed off to COX17 and then passed onto either SCO1, which then transfers copper to COX2 subunit of cytochrome oxidase, or COX11, which transfers copper to COX1 subunit of cytochrome oxidase. Copper exporter ATP7A/B exports copper out of the cell by translocating to plasma membrane when intracellular copper concentrations are high. Glycoprotein interacts with copper and regulates ECM remodeling. Cu, Copper; Copper-transporter 1, CTR1 (a major copper importer); CCS, Copper chaperone for superoxide dismutase; Cu/Zn-SOD, copper/zinc-superoxide dismutase; ATOX1, a copper chaperone, also known as HAH1; LOXs, lysyl oxidases; COX, cytochrome oxidase; SCO, synthesis of cytochrome c oxidase; ATP, copper transporting P-type ATPases [systemic copper absorption (ATP7A) and copper excretion (ATP7B)]; ECM, extracellular matrix.

Supplementation of dietary copper has been reported to reduce the severity of osteochondrosis and other developmental cartilage lesions, which may result from enhanced collagen cross-linking and increased collagen II synthesis (Knight et al., 1990; Hurtig et al., 1993; Yuan et al., 2011). The chondroprotection efficacy of copper may be attributable to the anti-catabolic effects of Cu^2+^, which abrogates the degradation of cartilage matrix proteoglycan via inhibition of nitric oxide release (Pasqualicchio et al., 1996). A recent study has reported that copper-incorporated bioactive glass-ceramics facilitated the regeneration of cartilage and osteochondral interface effectively, which was mediated in part through activated HIF-1 signaling and inhibited inflammatory response, representing a feasible approach for treating osteoarthritis associated with osteochondral defects (Lin R. et al., 2019). However, until now, the detailed mechanisms of interactions between copper and chondrocytes, as well as copper trafficking within chondrocytes remain elusive. To date, copper has been identified as a cofactor of several identified major cartilage formation-associated enzymes (Rucker et al., 1998; Heraud et al., 2002; Makris et al., 2013), however, cellular and molecular mechanisms underlying intracellular copper transportation in chondrocytes are not yet clearly understood. A cartilage matrix glycoprotein, a membrane-associated protein synthesized by chondrocytes, has been demonstrated to bind copper and exert some oxidase activity similar with ceruloplasmin, which may function as an important copper transporter in chondrocytes and a potential chondrogenic marker (Fife et al., 1986, 1993; Harris, 2000; Ranganathan et al., 2011; La Mendola et al., 2012; Ishihara et al., 2014; Linder, 2016; Magri et al., 2018). The proposed cellular model of copper intracellular transportation has been presented in Figure 3 according to current literature (Okado-Matsumoto and Fridovich, 2001; Carr et al., 2005; Horng et al., 2005; Turski and Thiele, 2009; Ohrvik and Thiele, 2014; Urso and Maffia, 2015; Miller et al., 2019; Shi et al., 2019), illustrating the routes of copper trafficking and how it functions within chondrocytes and during ECM remodeling.

**FIGURE 4:**
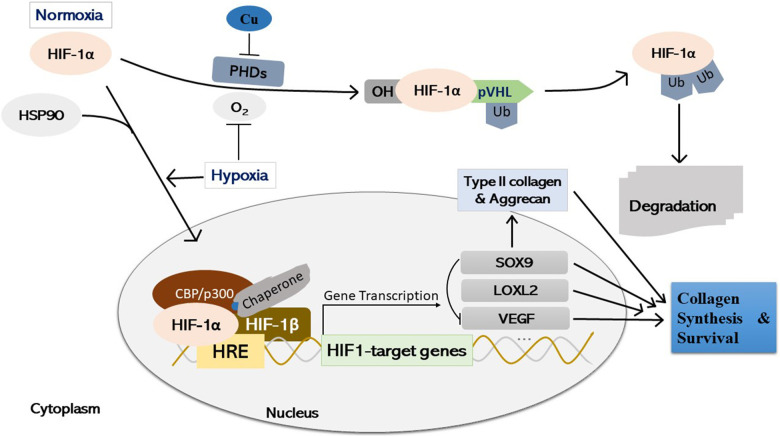
Schematic illustration of copper-mediated hypoxia-response element (HRE)-directed transcriptional fine-tuning of cartilage homeostasis-associated genes in chondrocytes. Under normoxia, HIF-1α undergoes PHDs-mediated prolyl hydroxylation, and prolyl OH HIF-1α is ligated by pVHL, an E3 ubiquitin ligase, and degraded by the proteasome finally. Copper stabilizes HIF-1α protein by inhibiting PHDs activity in an iron-independent manner. Under hypoxia or through interaction with HSP90, HIF-1α stabilizes and accumulates in the cell nucleus, where it forms a dimer with the HIF-1β subunit and a putative unidentified copper-chaperone. The dimer then forms a transcriptional complex with coactivator CBP/p300 through binding with HRE, regulating the expression of various downstream target genes, such as LOXL2, SOX9, and VEGF. Simultaneously, SOX9 is a negative regulator of VEGF, whilst the expression of SOX9 target genes (i.e., Type II collagen, and Aggrecan) is initiated, which is essential for cartilage synthesis and survival during both embryonic joint development and cartilage homeostasis. HIF-1α, hypoxia-inducible factor-1α; PHDs, prolyl hydroxylases; OH: enzymatic hydroxylation; pVHL, von Hippel-Lindau tumor suppressor protein; HRE, hypoxia-response element; Ub, ubiquitinated; SOX9, SRY (sex determining region Y)-box 9; HSP90, heat shock protein 90; LOXL2, lysyl oxidase-like 2; VEGF, vascular endothelial growth factor; Cu, copper.

Further, emerging evidence has indicated that copper stabilizes the HIF-1α protein through inhibiting prolyl hydroxylases-mediated prolyl hydroxylation in an iron-independent manner, which is required for transcriptional activation of HIF-1 of a series of target genes (Martin et al., 2005; Feng et al., 2009; Himoto et al., 2016; Liu et al., 2018; Chen et al., 2020), suggesting that appropriate copper levels may be required for normal cartilage function through regulation of HIF-1α transcriptional activity. However, detailed mechanisms of transcriptional processes initiating specific target genes expression of HIF-1 require further elucidation. Therefore, copper may modulate cartilage homeostasis through regulation of activity of HIF-1 transcription and chondrogenic-associated proteins and markers, such as LOXL2, and SOX9 (Robins et al., 2005; Amarilio et al., 2007; Schietke et al., 2010), as well as VEGF, which is essential for chondrocyte survival (Cramer et al., 2004; Maes et al., 2004, 2012) (Figure 4). Also, further extensive studies on regulation of HIF-1 expression (such as heat shock protein 90, HSP90), and specific targeting of certain transcriptional activation of certain HIF-1 target genes (Minet et al., 1999; Isaacs et al., 2002; Katschinski et al., 2004), as well as regulation of systemic copper metabolism are promising for potential therapeutic optimization (Isaacs et al., 2002; Kim et al., 2010).

### Potential Links Between LOX and Aging-Associated Cartilage Degeneration

Aging, which is characterized by a chronic and low-grade inflammation, also termed as age-associated inflammation, which has been referred to as ‘inflamm-aging’ (Franceschi and Campisi, 2014; Huang et al., 2019; Josephson et al., 2019), is also a risk factor of osteoarthritis (Greene and Loeser, 2015). Inflammation is a normal process in healthy individuals. Acute inflammation initiates the regenerative response (Kyritsis et al., 2012). Whilst chronic inflammation is likely to cause various diseases (Akhurst et al., 2005; Liu et al., 2019). The signaling pathways that are implicated in chronic inflammation include NF-κB (Roman-Blas and Jimenez, 2008; Bamborough et al., 2010), signal transducer and activator of transcription (STAT) (He and Karin, 2011), mitogen-activated protein kinases (MAPKS) (Thalhamer et al., 2008). Interestingly, a recent study reported that LOXL3-mediated deacetylation/deacetylimination abolished the transcription activity of STAT3, thereby inhibiting differentiation of naïve CD4^+^ T cells toward Th17/Treg cells (regulatory T cells) during inflammatory responses (Ma et al., 2017).

Aging-associated destruction of joints and cartilage degradation in osteoarthritis is correlated with changes in extracellular matrix of articular cartilage, such as cartilage ECM stiffness, and in the levels and solubility profiles of matrix crosslinks, especially pentosidine, as well as reduced thickness of cartilage, proteolysis, advanced glycation and calcification (Eyre et al., 1988; Pokharna et al., 1995; Lotz and Loeser, 2012). Notably, rejuvenation has emerged as a promising therapeutic regenerative approach for improvement or restoration of the self-repair capacity of injured or aging tissue and organ systems (Leung et al., 2006; Nelson et al., 2008; Luria and Chu, 2014; Sarkar et al., 2020), which has been proposed as a conversion into an embryonic-like state recapitulating many events during embryogenesis, including the reactivation of embryonic signature genes, and cytoskeletal/ECM components, and lineage specification (Vortkamp et al., 1998; Jankowski et al., 2009; Luo et al., 2009; Adam et al., 2015; Caldwell and Wang, 2015; Hu et al., 2017; Ransom et al., 2018; Feng et al., 2019; Lin W. et al., 2019; Miao et al., 2019). Consistently, the process of cartilage repair has been considered as recapitulation of various events during developmental morphogenesis. Chondrocytes in osteoarthritic articular cartilage usually undergoes a gradual dissolution of anisotropic organization along with re-expression of phenotypic biomarkers of immature cartilage, so tissue maturation is a potential approach for restoration of normal structure and function (Caterson et al., 1990; Khan et al., 2008; Hunziker, 2009; Jiang et al., 2015; Zhang et al., 2017).

Interestingly, copper is also involved in inflammatory responses, including both innate and adaptive immunity (Percival, 1998; Failla, 2003). Increasing evidence has indicated the potential link between copper metabolic disorder and aging-related diseases, such as aging-induced cartilage degradation and dysfunction (Yazar et al., 2005; Lotz and Loeser, 2012; Tao et al., 2019). And previous studies have demonstrated the vital roles of LOX in normal chondrocyte function (Sanada et al., 1978; Ahsan et al., 1999), which may be correlated with the pathogenesis of aging-associated osteoarthritis (Pokharna et al., 1995). Moreover, LOXL1 is expressed in major organs in late fetal and neonatal mice, but it generally diminishes in aging animals, which may be associated with aortic fragility resulting from abnormal remodeling of collagen and elastic fibers (Hayashi et al., 2004; Behmoaras et al., 2008). The decreased expression of LOXs may attribute to reduction of HIF-1 activity in aging organisms (Rivard et al., 2000; Ceradini et al., 2004). Meanwhile, LOXL2 has recently been demonstrated as a potential chondroprotective factor in aging related joint osteoarthritis, mainly through inducing anabolic gene expression and attenuating catabolic genes (Bais and Goldring, 2017; Tashkandi et al., 2019). Thus, restoration of collagen and elastic fiber synthesis in juvenile ECM components though regulation of signaling pathways governing LOX expression may become a promising therapeutic approach for amelioration of aging-associated cartilage degeneration and enhanced cartilage regeneration.

Meanwhile, changes in cell-ECM interactions are important features of aging phenomenon (Sun et al., 2011). During the progression of aging-associated degeneration diseases, altered cell fate of adult stem cells, or dysfunction of terminally differentiated mature cells occurs, which may result from the changed ECM niche modified with aging-related proteins and reduced expression of ECM-synthesis-associated proteins (Goupille et al., 1998; Chan et al., 2006; Sakai et al., 2012; Wang et al., 2015; Jeon et al., 2017; Zhang Y. et al., 2018; Patil et al., 2019), which may be correlated with the LOX family members. Therefore, reverting aging-associated genes in ECM may become an important strategy for joint rejuvenation (Chan et al., 2006; Fuoco et al., 2014; Li et al., 2014; Sun et al., 2011; Wang et al., 2019; Zhou J. et al., 2019). Modulation of expression of LOXs family members through transcriptional regulation of HIF-1 may become promising therapeutic approaches for treating aging-induced cartilage degeneration as potential rejuvenating therapies.

## Concluding Remarks

In summary, LOXs play pivotal roles in maintenance of cartilage function and chondrogenesis. Therapeutic modulation of LOX activity and expression selectively targeting copper-mediated hypoxia-responsive signaling pathways is promising for cartilage repair and OA attenuation. Meanwhile, further extensive basic and preclinical research is warranted for potential translational application of the LOX family in tissue-engineered neocartilage in tissue engineering and regenerative medicine in the future. Specific and moderate manipulation of activities of LOXs and transcriptional regulation of hypoxia-responsive transcription factors through copper bioavailability modulation or continuous hypoxia-conditioning may become effective interventions for enhanced cartilage regeneration, as well as promising rejuvenation therapeutics, which may exert further therapeutic implications in the upcoming clinical arena.

## Author Contributions

WL and LX made literature review and contributed equally. GL conceptualized the study and critically revised the manuscript. All authors read and approved the final manuscript.

## Conflict of Interest

The authors declare that the research was conducted in the absence of any commercial or financial relationships that could be construed as a potential conflict of interest.
