# Corrigendum: Molecular Insights Into Lysyl Oxidases in Cartilage Regeneration and Rejuvenation

**DOI:** 10.3389/fbioe.2020.598323

**Published:** 2020-10-26

**Authors:** Weiping Lin, Liangliang Xu, Gang Li

**Affiliations:** ^1^Department of Orthopaedics and Traumatology, Stem Cells and Regenerative Medicine Laboratory, Li Ka Shing Institute of Health Sciences, The Chinese University of Hong Kong, Prince of Wales Hospital, Hong Kong, China; ^2^The First Affiliated Hospital of Guangzhou University of Chinese Medicine, Lingnan Medical Research Center, Guangzhou University of Chinese Medicine, Guangzhou, China; ^3^MOE Key Laboratory for Regenerative Medicine, School of Biomedical Sciences, The Chinese University of Hong Kong, Hong Kong, China

**Keywords:** lysyl oxidase, cartilage, hypoxia-inducible factor, copper, transcription activity, regeneration, rejuvenation

In the original article, there was a mistake in Figure 4 as published. The corrected Figure 4 and figure legend appear below.

**Figure 4:**
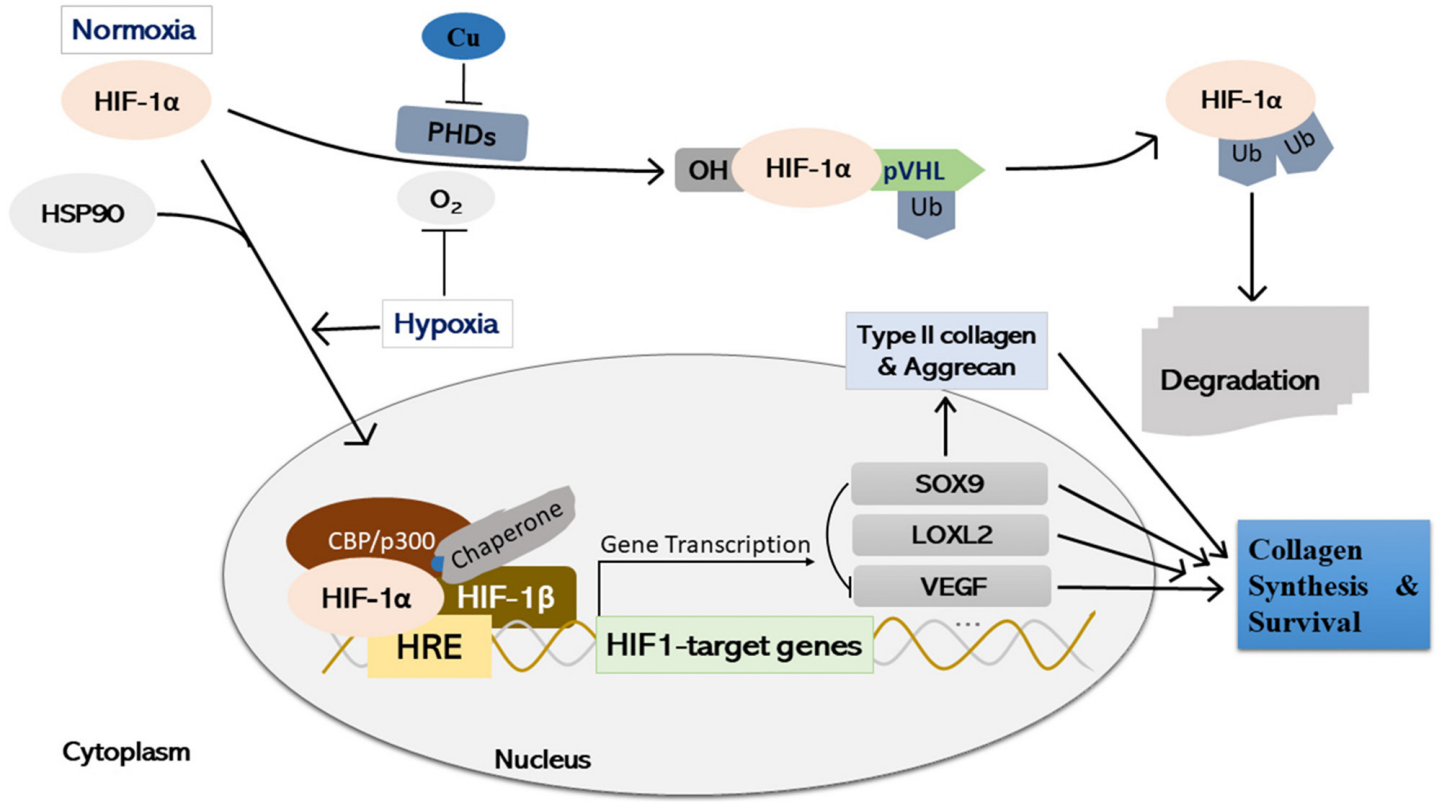
Schematic illustration of copper-mediated hypoxia-response element (HRE)-directed transcriptional fine-tuning of cartilage homeostasis-associated genes in chondrocytes. Under normoxia, HIF-1α undergoes PHDs-mediated prolyl hydroxylation, and prolyl OH HIF-1α is ligated by pVHL, an E3 ubiquitin ligase, and degraded by the proteasome finally. Copper stabilizes HIF-1α protein by inhibiting PHDs activity in an iron-independent manner. Under hypoxia or through interaction with HSP90, HIF-1α stabilizes and accumulates in the cell nucleus, where it forms a dimer with the HIF-1β subunit and a putative unidentified copper-chaperone. The dimer then forms a transcriptional complex with coactivator CBP/p300 through binding with HRE, regulating the expression of various downstream target genes, such as LOXL2, SOX9, and VEGF. Simultaneously, SOX9 is a negative regulator of VEGF, whilst the expression of SOX9 target genes (i.e., Type II collagen, and Aggrecan) is initiated, which is essential for cartilage synthesis and survival during both embryonic joint development and cartilage homeostasis. HIF-1α, hypoxia-inducible factor-1α; PHDs, prolyl hydroxylases; OH: enzymatic hydroxylation; pVHL, von Hippel-Lindau tumor suppressor protein; HRE, hypoxia-response element; Ub, ubiquitinated; SOX9, SRY (sex determining region Y)-box 9; HSP90, heat shock protein 90; LOXL2, lysyl oxidase-like 2; VEGF, vascular endothelial growth factor; Cu, copper.

The authors apologize for this error and state that they do not change the scientific conclusions of the article in any way. The original article has been updated.

